# Mechanosensitive extrusion of Enterovirus A71-infected cells from colonic organoids

**DOI:** 10.1038/s41564-023-01339-5

**Published:** 2023-03-13

**Authors:** Jasmine Moshiri, Ailsa R. Craven, Sara B. Mixon, Manuel R. Amieva, Karla Kirkegaard

**Affiliations:** 1 Department of Microbiology and Immunology, Stanford University, Stanford, CA USA; 2 Department of Genetics, Stanford University, Stanford, CA USA; 3 Department of Pediatrics, Stanford University, Stanford, CA USA

**Keywords:** Viral transmission, Mechanisms of disease, Infection

## Abstract

Enterovirus A71 causes severe disease upon systemic infection, sometimes leading to life-threatening neurological dysfunction. However, in most cases infection is asymptomatic and limited to the gastrointestinal tract, where virus is amplified for transmission. Picornaviruses have previously been shown to exit infected cells via either cell lysis or secretion of vesicles. Here we report that entire Enterovirus A71-infected cells are specifically extruded from the apical surface of differentiated human colon organoids, as observed by confocal microscopy. Differential sensitivity to chemical and peptide inhibitors demonstrated that extrusion of virus-infected cells is dependent on force sensing via mechanosensitive ion channels rather than apoptotic cell death. When isolated and used as inoculum, intact virus-containing extruded cells can initiate new infections. In contrast, when mechanical force sensing is inhibited, large amounts of free virus are released. Thus, extrusion of live, virus-infected cells from intact epithelial tissue is likely to benefit both the integrity of host tissues and the protected spread of this faecal–oral pathogen within and between hosts.

## Abstract (web summary)

Enterovirus-infected cells are extruded from the apical surface of differentiated human colon organoids by a mechanical-force-dependent mechanism that preserves the uninfected host epithelium.

## Main

The question of how progeny viruses leave an infected cell or tissue is vital to our understanding of viral spread throughout an infected host and between hosts. For decades, picornaviruses, as non-enveloped or ‘naked’ viruses, were thought to transmit strictly lytically, through dramatic rupture of the infected cell. Lytic release of picornaviruses from standard tissue culture cell lines results in widespread dispersal of virions to initiate subsequent rounds of infection. However, non-lytic spread has been demonstrated for several picornaviruses, during which the viruses appropriate intracellular membranes to facilitate their release from intact cells, cloaked within extracellular vesicles^1–4^. One consequence of this transmission strategy is that, instead of the dispersive spread of single viral particles, viruses are transmitted en bloc within membranous packets^5^.

Epithelial organoid models are exciting tools to examine tissue-specific responses to pathogenic insults^6,7^. Adult stem cell-derived 3D spherical organoids can be differentiated to recapitulate the diversity of cell types present in native tissues^8^. Organoids typically grow with their apical surfaces facing interior lumenal compartments. 2D polarized monolayers or organoids that were mechanically sheared to allow apical access of Enterovirus A71 (EV-A71) have been used to model EV-A71 infection of the gastrointestinal epithelium^9–11^. Recently, methods to invert organoid topology have been developed to present the apical surface of gastrointestinal epithelia to enteric pathogens directly while preserving epithelial integrity^12,13^. In this Article, these apical-out organoids are used to monitor the infection of epithelial cells by EV-A71 and subsequent mechanisms of viral spread in this tissue.

## Results

### Susceptibility of apical-out colonoids to EV-A71 infection

**Fig. 1:**
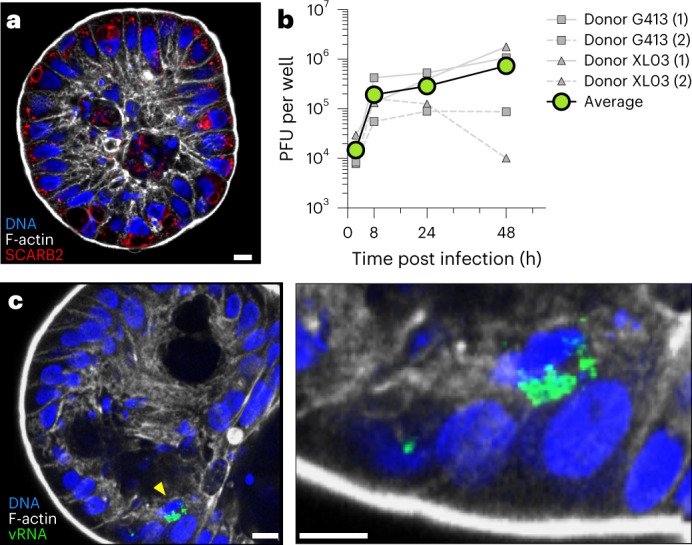
Susceptibility of apical-out colon organoids to EV-A71 virus. **a**, Apical-out, differentiated colon epithelial organoids express the EV-A71 receptor SCARB2 (red), localizing to intracellular membranes. The integrity of the apical actin (white) microvillus brush border is visible. **b**, Colon organoids were infected with EV-A71. Viral titres were monitored over time by plaque assay. Data from four independent experiments using two different colonoid donors are shown. **c**, EV-A71-infected cells were observed by immunofluorescence assay following staining for double-stranded vRNA after 48 h of infection. Right: increased magnification of infected cell denoted by yellow arrowhead in the left panel. Scale bars, 10 μm. Source data

We were interested in using apical-out gastrointestinal epithelial organoids as a model of EV-A71 infection in which epithelial barrier integrity is maintained. We chose to use organoids derived from colonic tissue (colonoids), due to the proximity of the colon to viral exit from an infected host, to better understand EV-A71 transmission. Colonoids derived from adult human crypt tissue were grown on basement membrane scaffolds in the presence of stem factors, including WNT and R-spondin, generating spheroids of stem cells with their basolateral surface facing outwards and their lumenal, apical surfaces facing inwards. Five days before infection, colonoids were removed from the basement membrane scaffold and kept in suspension culture in the absence of matrix to induce the reversal of organoid topology so that the apical surfaces were on the organoid exterior (apical-out)^12^. At the same time, medium to induce cell differentiation was applied. In these apical-out human colonoids, we monitored the organization of the actin cytoskeleton and EV-A71 receptor SCARB2 (Fig. 1a). The apical surface of well-differentiated and polarized colonoids contains brush border microvilli, which are easily identifiable with F-actin staining as a thick, actin-rich layer. SCARB2 is an integral lysosomal membrane protein that cycles to the apical surface of polarized monolayers^14,15^ and was abundantly expressed in these apical-out colonoids (Fig. 1a).

Viral growth curves beginning immediately after infection showed that EV-A71 could productively infect differentiated apical-out colonoids from two different human donors, with virus accumulation in the culture continuing up to 48 h after infection (Fig. 1b). At that time, infected colonoids were fixed, stained and examined by confocal microscopy for the presence of double-stranded viral RNA (vRNA), which is apparent in infected cells after fixation, to identify vRNA replication complexes. Punctate juxtanuclear and cytoplasmic staining patterns indicative of vRNA replication complexes were readily observed (Fig. 1c). Curiously, the majority of infected cells were solitary amidst their uninfected neighbours.

### EV-A71-infected cells are extruded from colonoids

**Fig. 2:**
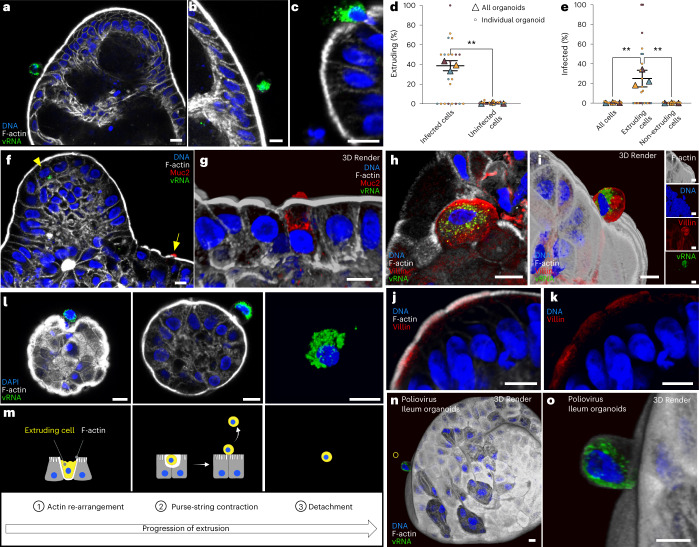
Specific extrusion of EV-A71-infected cells from colonoids. **a**–**c**, Infected colonoids were fixed at 48 h post infection and stained for double-stranded vRNA. **d**, Infected cells were extruded from colonoids with higher frequency than uninfected cells. The percentages of infected or uninfected cells extruding from an individual organoid are shown as small circles. The proportion of cells extruding across all organoids in each experiment are shown as triangles of the same colour as the individual colonoids. At least ten organoids were quantified per experiment: **P < 0.01; paired, two-tailed *t*-test, *N* = 3 experiments. **e**, Percentages of infected extruding and non-extruding cells were measured similarly. Of the cells that were extruding, a higher percentage was infected. **P < 0.01; repeated measures one-way ANOVA with Tukey’s multiple comparisons test, *N* = 3 experiments. In **d** and **e**, raw cell numbers represented in the graphs are displayed in Supplementary Table 1. **f**, EV-A71-infected colonoids were stained for Muc2 expression, a marker for goblet cells. Arrowhead indicates infected cell. Arrow indicates goblet cell shown in **g**. **g**, Muc2-expressing goblet cell. **h**,**i**, Villin expression identifies colonocytes. Villin localizes apically, overlapping with the actin-rich microvillus brush border. Distinct individual cells are shown. **j**,**k**, EV-A71-infected colonoids were stained for Villin expression. **l**, Representative extruding infected cells are shown. **m**, Stages of canonical cell extrusion in uninfected epithelia are depicted. **n**,**o**, Cells infected with poliovirus Type 1 (Mahoney) were observed extruding from ileum organoids by immunofluorescence. **o** shows an individual cell highlighted in **n**. Scale bars, 10 μm. In **d** and **e**, horizontal and error bars represent mean ± s.d. Source data

The lack of observable viral spread, even as viral yield was increasing, could be rationalized following careful inspection of the apical surfaces of the infected colonoids. We were surprised by the frequent observation of virus-infected cells that appeared to be extruding from intact colonoids (Fig. 2a–c). In healthy gastrointestinal epithelia, whole-cell extrusion maintains homeostatic cell numbers by facilitating removal of existing cells to match the rate of stem cell expansion in the crypts. In fact, so many cells are extruded that the average lifespan of a gastrointestinal epithelial cell is just 2–5 days^16,17^. To determine whether the frequencies at which EV-A71-infected cell extrusion exceeded those of uninfected cells, we quantified how frequently both infected and uninfected cells underwent extrusion from the same EV-A71-infected colonoids (Fig. 2d,e and Supplementary Table 1). Infected colonoids were fixed, stained and examined by confocal microscopy. Individual cells were categorized as (1) infected or uninfected and (2) extruding or non-extruding. A cell was defined as extruding from an organoid if its nucleus had crossed the apical border of the organoid, visualized by staining the cortical actin with phalloidin. At the 48 h timepoint examined, 40% of the infected cells were extruding, compared with only 1% of the uninfected cells (Fig. 2d and Supplementary Table 1). Similarly, although infected cells constituted only 0.4% of the cells within the organoids, they constituted 25% of the extruding cells (Fig. 2e and Supplementary Table 1). These data demonstrate that infected cells are extruded from colonoids at frequencies significantly higher than expected by random chance.

To identify the permissive cell type in the colonoids, we performed immunostaining with antibodies targeting Villin1 and Muc2, expressed by absorptive colonocytes and goblet cells, respectively (Fig. 2f–k). Both cell types have been previously implicated in EV-A71 infection in the gastrointestinal epithelium^10,11^. Our observations suggest that absorptive colonocytes, rather than goblet cells, were the primarily infected cell type in this model. This finding does not preclude infection of goblet cells in epithelia in which goblet cells are more plentiful.

We observed infected cells in several distinct states reminiscent of the stages of canonical whole-cell extrusion (Fig. 2l,m). Cell extrusion is a highly coordinated process that allows the removal of undesirable cells while maintaining the integrity of the epithelial barrier^18,19^. During canonical extrusion, cells fated for extrusion and their neighbouring cells re-arrange their cytoskeletons to surround the base of the extruding cells; these actin–myosin rings contract apically to squeeze out the cells being extruded by what has been termed a ‘purse-string’ mechanism (Fig. 2n)^20^. At the same time, tight junctions re-form between the new neighbour cells below the extruding cells to maintain epithelial integrity^21,22^. Cell displacement and formation of new neighbours occur over a period of approximately 40 min. Extruded cells cling to the epithelium for an additional 40 min before detaching from their neighbours and floating away^20,23^. We observed infected cells in states consistent with both early and late stages of extrusion, as shown in Fig. 2l. On the left, an infected cell can be seen embedded within an organoid, surrounded by an actin layer condensing beneath the infected cell. The middle image shows an extruded cell still in contact with a re-formed, intact apical surface. The fully detached infected cell on the right was observed floating in suspension alongside infected organoids.

**Extended Data Fig. 1:**
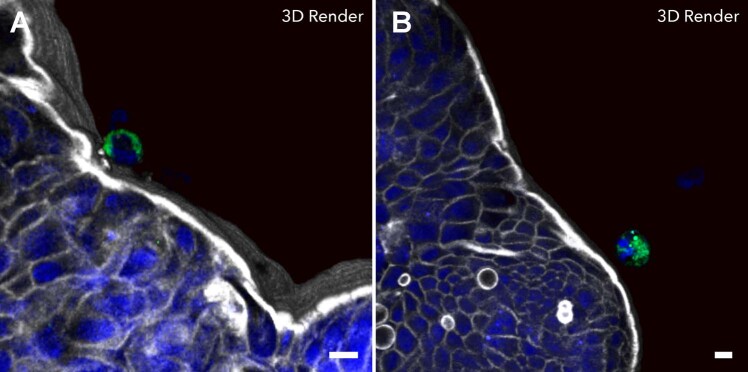
Poliovirus-infected cells extrude from infected colon organoids. Cells infected with poliovirus Type 1 (Mahoney) were observed extruding from organoids by immunofluorescence. (**a-b**) Colon organoids infected with an MOI = 1 PFU/cell for 42 h. MOI was determined by viral titer on HeLa cells. Scale bars equal 10 μm.

**Extended Data Fig. 2:**
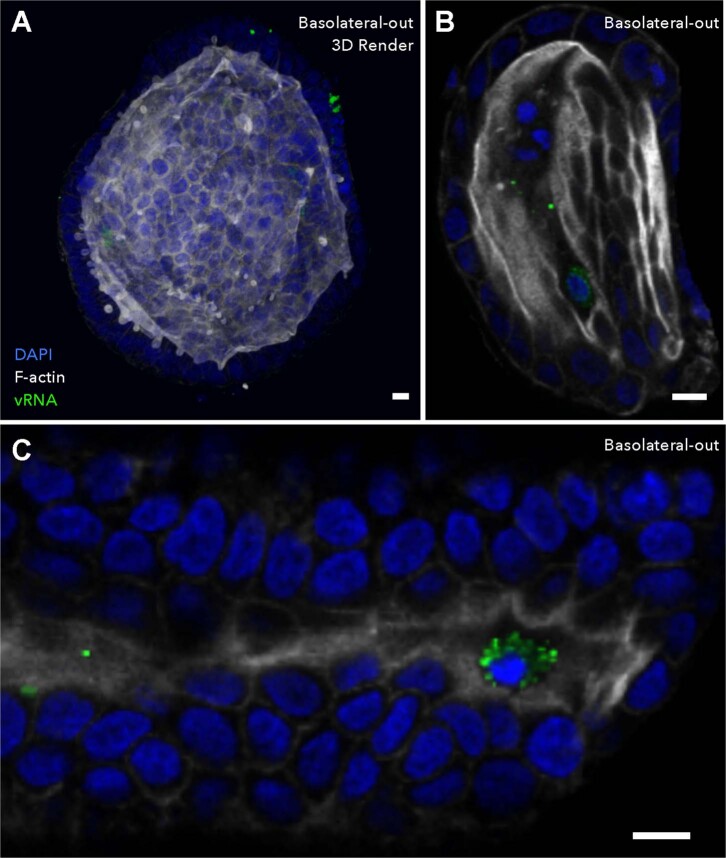
Apical extrusion of EV-A71 infected cells occurs regardless of organoid polarity. Differentiated colonoids with basolateral-out organoid polarity were infected with EV-A71 and visualized by immunofluorescence at 8 hours post-infection. (**a**) Strong F-actin staining of the apical microvillus brush border was observed on the interior lumen of infected colonoids. Virus infected cells can be observed both within the epithelial layer, as well as within the apical lumen. (**b, c**) EV-A71 infected cells were apically extruded into the interior lumen of basolateral-out colonoids. Scale bars equal 10 μm.

To investigate whether the phenomenon of infected cell extrusion was specific to EV-A71, we examined cells infected with poliovirus, a member of the related Enterovirus C species. In organoids derived from ileal tissue of the small intestine as well as colonic tissue, extruding poliovirus-infected cells were readily observed (Fig. 2n,o and Extended Data Fig. 1). We suggest that multiple enteroviruses instigate the ejection of infected cells from gastrointestinal organoids by whole-cell extrusion. We additionally questioned whether EV-A71-infected cell extrusion was specific to the epithelial architecture of apical-out organoids. However, extrusion of infected cells was readily observed in the contrasting basolateral-out colonoids (Extended Data Fig. 2).

**Extended Data Fig. 3:**
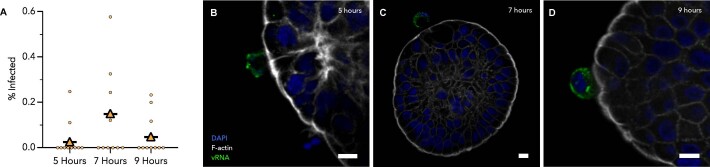
Infected cells accumulate in organoids after 7 h infection and diminish after 9 h. (**a**) Infected organoids were fixed at timepoints over the first cycle of infection and the number of infected and uninfected cells were enumerated using fluorescence microscopy. Each dot represents the proportion of infected cells within an individual organoid; triangles represent values averaged across all organoids for each timepoint. One experiment with N = 10 organoids quantified per timepoint. A peak in the proportion of infected cells within organoids emerged at 7 hpi and was reduced after 9 hpi. Raw cell numbers represented in this graph are displayed in Supplementary Table 2. (**b–d**) Representative infected cells from each timepoint. Extruding infected cells contain similarly abundant vRNA signal regardless of timepoint visualized. Scale bars equal 10 μm. Source data

To characterize the timing of infected cell extrusion, we visualized infected cells within colonoids during the first round of infection (Extended Data Fig. 3 and Supplementary Table 2). We observed that infected cells can be extruded as early as 5 h and as late as 9 h post infection, and that extruding infected cells at all timepoints contained abundant vRNA. Therefore, cell-to-cell differences in infection kinetics^24^ probably contribute to the timing of extrusion. The number of infected cells in colonoids increased significantly from 5 h to 7 h after infection, but substantially reduced afterwards, even though the amount of infectious virus in the entire culture continued to rise (Fig. 1b). That infected cells are predominately shed between 7 h and 9 h after infection is consistent with the lack of observed cell-to-cell transmission within the colonoids.

### Extruded EV-A71-infected cells are largely not apoptotic

**Fig. 3:**
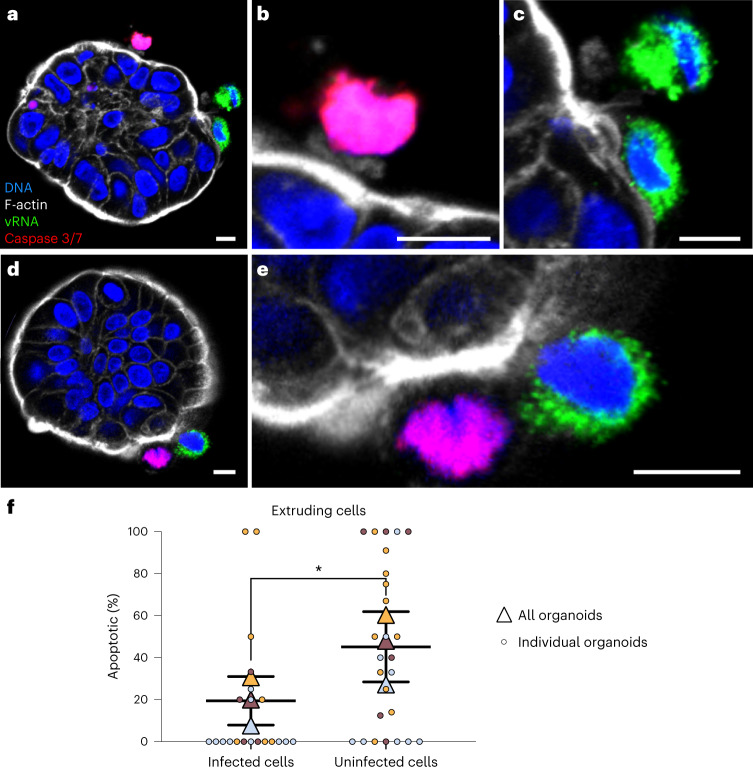
EV-A71-infected cell extrusion is not driven by apoptosis. Infected colonoids were visualized by immunofluorescence confocal microscopy after 48 h of infection. Caspase 3 and 7 activity was visualized using a fluorogenic substrate. **a**, Individual infected organoids with extruding cells. **b**, Increased magnification of single uninfected, apoptotic extruding cell in **a**. **c**, Increased magnification of two infected, non-apoptotic extruding cells in **a**. **d**, Individual infected organoids with extruding cells. **e**, Magnification of two extruding cells in **d**. **f**, Proportions of infected extruding and uninfected extruding cells that were apoptotic were quantified, with overall values for each experiment shown as triangles. Each colour represents an independent experiment, with measurements for each individual organoid shown as small circles. *P < 0.05; paired, two-tailed *t*-test, *N* = 3; horizontal and error bars represent mean ± s.d. Raw cell numbers represented in this graph are displayed in Supplementary Table 3. Scale bars, 10 µm. Source data

Extrusion of apoptotic cells from intact epithelia was originally described as a means to remove dying cells without compromising the epithelial barrier^20^. Given that EV-A71 infection can trigger apoptosis in several cell types^25–28^, we tested whether apoptotic signalling triggers extrusion of infected cells in colonoids. We utilized a fluorogenic substrate (CellEvent, ThermoFisher) to visualize cells that expressed active caspases 3 and 7. Infected organoids were incubated with substrate, fixed, stained for double-stranded RNA and examined by confocal microscopy (Fig. 3a–e). Approximately half of the uninfected extruded cells were caspase 3/7 positive, consistent with the normal functioning of cell extrusion in intestinal epithelia^29^. However, a significantly lower fraction of infected, extruding cells were caspase 3/7 positive (Fig. 3f and Supplementary Table 3). Furthermore, the nuclei in infected extruding cells were usually intact and did not display the condensed and fragmented nuclei characteristic of apoptotic cells. These data argue that apoptotic stress does not cause infected cell extrusion.

### Mechanosensing ion channel activity mediates EV-A71-infected cell extrusion

**Fig. 4:**
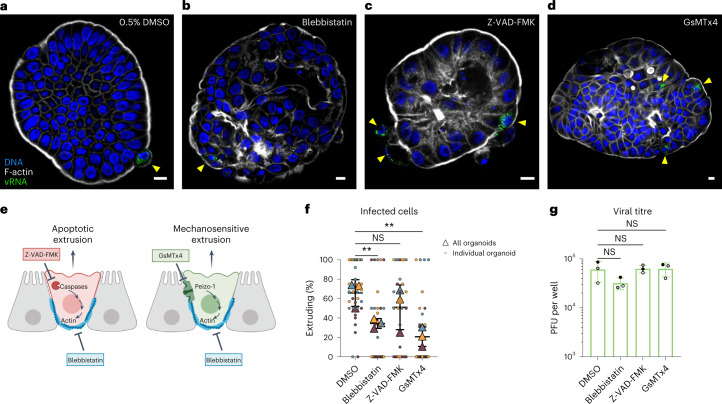
Mechanosensitive signalling promotes infected cell extrusion. EV-A71-infected organoids were exposed to compounds capable of inhibiting cellular factors implicated in different mechanisms of extrusion. **a**–**d**, Infected organoids were exposed to 0.5% DMSO vehicle control (**a**), 50 μM *para*-nitro-blebbistatin (**b**), 100 μM Z-VAD-FMK (**c**) or 20 μM GsMTx4 (**d**). Infected cells in organoids were visually inspected by confocal microscopy. Yellow arrowheads indicate infected cells in representative organoids. Scale bars, 10 μm. **e**, Blebbistatin inhibits both apoptotic and mechanosensitive extrusions, Z-VAD-FMK inhibits only apoptotic extrusion and GsMTx4 inhibits only mechanosensitive extrusion. **f**, The percentage of infected cells undergoing extrusion after 7 h of infection was enumerated. Each colour shows an independent experiment. Overall proportion of infected cells extruding per experiment shown as triangles, with measurements for each organoid shown as small circles. **P < 0.01; NS, not significant; repeated measures one-way ANOVA with Dunnett’s multiple comparisons test, *N* = 3. **g**, Viral titres quantified at 7 h post infection from infected suspension organoid cultures show no significant effects of drug treatments on virus yield. Repeated measures one-way ANOVA with Dunnett’s multiple comparisons test, *N* = 3 independent infections. In **f** and **g**, horizontal and error bars represent mean ± s.d. Source data

In addition to apoptotic or pyroptotic cell death, cell extrusion from the gastrointestinal epithelium can be triggered by mechanical forces on cells due to overcrowding^30,31^. Although it plays no role in extrusion of apoptotic cells, the mechanosensitive ion channel Piezo1 senses and responds to cell crowding stress, triggering cell extrusion^29^. We hypothesized that alteration of the biomechanical properties of infected cells may be sensed by Piezo1, leading to force-dependent extrusion of infected cells. To test this hypothesis, we treated infected colonoids with GsMTx4, a spider venom peptide that inhibits the activity of mechanosensitive ion channels including Piezo1 (refs. ^32,33^). We also evaluated the effect of Z-VAD-FMK, a pan-caspase inhibitor known to reduce apoptotic cell extrusion^23^. Finally, given that actin-myosin re-arrangement is critical for cell extrusion regardless of initial trigger, we tested the effect of myosin II inhibitor *para*-nitro-Blebbistatin^34^ as a positive control for inhibition of all cell extrusion mechanisms^35,36^ (Fig. 4e).

Following 2 h of infection with EV-A71, colonoids were treated with compounds above for the remainder of a single cycle of infection (Fig. 4a–d). The proportion of infected cells extruding from colonoids was quantified under each condition (Fig. 4f). As expected, the percentage of infected cells extruding from organoids was reduced in the presence of blebbistatin and unaffected by Z-VAD-FMK, which inhibits only apoptotic extrusion. However, we observed a striking reduction in the percentage of infected cells undergoing extrusion in the presence of mechanosensitive ion channel inhibitor GsMTx4 (Fig. 4f). To exclude the possibility that this was due to inhibition of viral growth, we evaluated the effect of all compounds on viral yield, none of which was changed (Fig. 4g). These results argue that it is the force-sensing activity of mechanosensitive ion channels targeted by GsMTx4 that is crucial for the elimination of live, EV-A71-infected cells.

### The fate of extruded infected cells

**Fig. 5:**
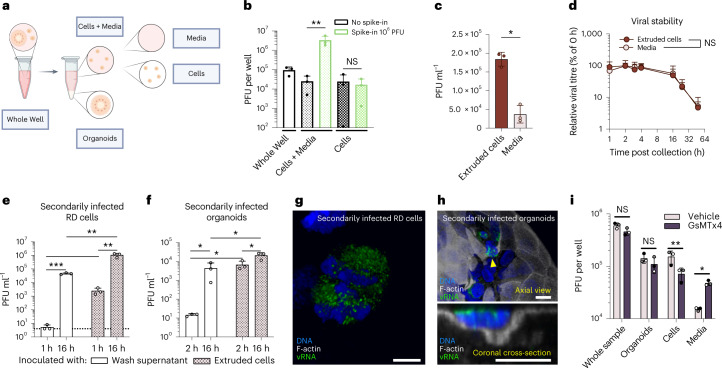
Extruded cells can infect cultured cells and organoids. **a**, EV-A71-infected colonoid cultures were collected at 8 h post infection, and components were collected by differential sedimentation. **b**, To assess effectiveness of washing extruded cells, 10^6^ PFU exogenous free virus was spiked-in to Cells + Media samples. Fractions including Whole Well (blank bars), Cells + Media (small dotted bars) and Cells (large-dotted bars) were subjected to freeze–thaw and plaque assay. Repeated-measures one-way ANOVA with the Holm–Šídák multiple comparisons test. NS, not significant. **c**, Distribution of virus in Cells and in Media. Ratio paired, two-tailed *t*-test. **d**, Stability of virus from Cells and Media fractions. Collected fractions were incubated at 37 °C for the times indicated, subjected to repetitive freeze–thaw and subsequent plaque assay. Amounts of virus are normalized to values from **c** before incubation. Two-way ANOVA with Geisser–Greenhouse correction. **e**, Intact extruded Cells fractions and free virus from the final wash of Cells were used to infect RD cell monolayers. Viral titres in the infected RD cells were measured 1 h and 16 h after initiating secondary infection. Ratio paired (cells–cells; wash–wash) and unpaired (wash–cells), two-tailed *t*-tests. Dashed line indicates limit of detection. **f**, Cells fractions and free virus from the final wash of Cells were used to infect new colonoids. After 2 h and 16 h, the amount of virus in Whole Well fractions was determined by plaque assay. Statistic testing as described in **e**. **g**,**h**, Confocal microscopy of secondarily infected RD cells (**g**) and organoids inoculated with Cells after 16 h (**h**). Scale bars, 10 μm. **h**, Secondarily infected organoid with several infected cells. Bottom: orthogonal cross-section through the secondarily infected, extruding cell indicated by yellow arrowhead. **i**, EV-A71-infected colonoid cultures were treated with GsMTx4 or vehicle, and fractions were collected after 8 h. While GsMTx4 treatment reduced the amount of infectious virus in extruded cells, the amount of infectious free virus in the media increased. Multiple paired, two-tailed *t*-tests with the Holm–Šídák correction for multiple comparisons. In **b**–**f** and **i**, one representative experiment is shown with independent infections performed in triplicate; *P < 0.05, **P < 0.01, ***P < 0.001; data represented are mean ± s.d. Source data

To determine whether the extruded, infected cells could provide a source of viral spread within the gastrointestinal tract, extruded cells (Cells) were collected by differential sedimentation (Methods) and the amount of virus within them was determined. To confirm that washing steps efficiently removed cell-free virus from the Cells fraction, 10^6^ plaque-forming units of exogenous virus were spiked into to a set of (Cells + Media) samples. The spiked-in virus greatly increased the viral titre in Cells + Media samples (Fig. 5b). However, the viral titre in the Cells was unchanged by the spike-in of exogenous virus, demonstrating that the washes successfully eliminated potential contaminating free virus. Additionally, as shown in Fig. 5c, there was significantly more infectious virus in the extruded Cells fractions than in cell-free Media.

**Extended Data Fig. 4:**
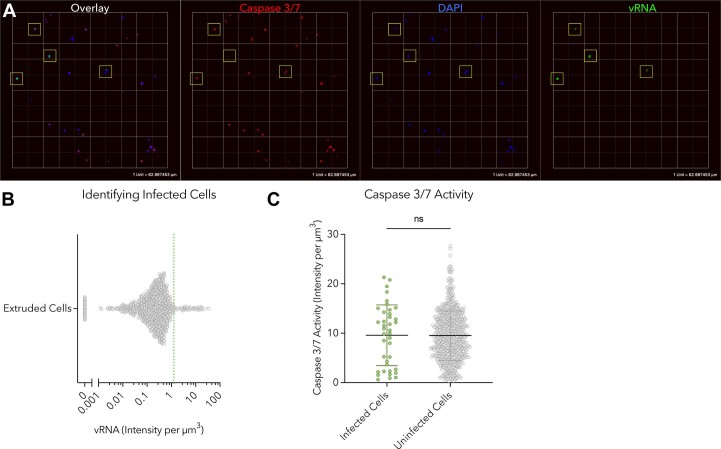
Extruded cells undergo anoikis by 48 hpi. Cells that had fully extruded from infected colonoids in the presence of the fluorogenic caspase 3/7 substrate CellEvent were collected at 48 hpi, fixed, and examined by fluorescence microscopy at 20x magnification. (**a**) 3D renderings of imaged cells were visualized using Volocity image analysis software. Infected cells are surrounded by yellow boxes. (**b**) Individual cells were identified from 20 unique fields of view as in A using computational measurement tools in Volocity. Data were collected from N = 743 individual cells. Cells with pixel intensities greater than 1.5 per cubic micron in the vRNA channel were identified as infected; N = 39 infected cells, 5.2% of total. (**c**) The caspase 3/7 activity of cells defined in B were compared. Caspase activity in infected cells was comparable to that of uninfected cells; unpaired, two-sided t-test; data presented as mean ± SD; N = 39 infected cells; N = 704 uninfected cells. Source data

To determine how long the extruded cells remained alive after extrusion, we monitored the apoptotic state of Cells after 48 h. At that timepoint, the majority of both infected and uninfected extruded cells were caspase 3/7 positive (Extended Data Fig. 4), suggesting that cells extruded as a result of viral infection eventually undergo detachment-induced apoptotic cell death. To test whether residence within these dying, extruded cells damaged the resident virions, Cell and Media preparations shown in Fig. 5c were incubated at 37 °C. All samples were subjected to repetitive freeze–thaw to lyse cells before plaque assay. Over a 48 h time course, virus residing within Cells retained stability equally as well as free virus in Media (Fig. 5d). Therefore, infected cells expelled from colonic epithelia contain stable, infectious virus.

To elucidate whether extruded cells are themselves infectious, Cells fractions were prepared from infected colonoids and used as inocula for secondary infections (Fig. 5e,f). Viral growth in both secondarily infected rhabdomyosarcoma (RD) cell monolayers and previously uninfected colonoids was examined by comparing titres immediately after infection and after 16 h of infection. As a control for effective isolation of Cells from any remaining free virus, supernatants from the last of three washes were also used as inocula. In cultures infected with cells extruded from previously infected organoids, abundant virus was present immediately after infection (Fig. 5e,f) and significant increases were observed upon incubation. Importantly, after 16 h the quantity of virus in cultures infected with extruded cells was significantly higher than that in cultures infected with wash supernatant, indicating that the presence of extruded cells, rather than residual cell-free virus, was responsible for the high viral loads. Confocal microscopy confirmed the presence of infected cells within secondarily infected RD monolayers and colonoids (Fig. 5g,h). Therefore, extruded, virus-containing cells are infectious to both RD monolayers and apical-out, differentiated colonoids.

### The fate of infected cells retained within colonoids

**Extended Data Fig. 5:**
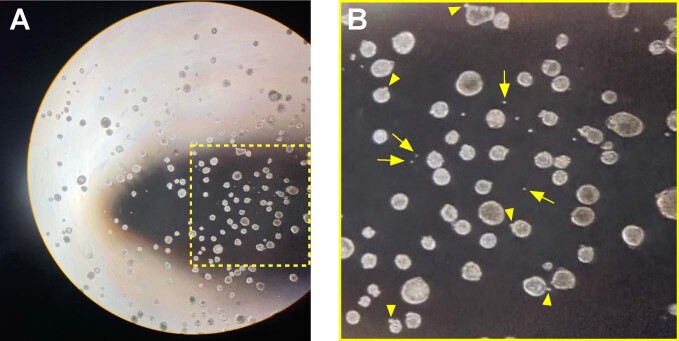
Examination of the Organoid fraction of suspension organoid cultures. Following fractionation of organoid cultures, all fractions were visually examined on a tissue culture microscope to assess collection of target populations. (**a**) Visual examination of the Organoid fraction at 40x magnification confirms that organoids are collected with gravity pellet (1 x g). (**b**) Region highlighted in A. Single cells were also present at low abundance in the organoid fraction (arrows indicate examples), potentially a result of ongoing cell extrusion from organoids during fractionation (arrowheads).

To determine the fate of the viruses and cells retained in the colonoid epithelial layer when extrusion was inhibited, colonoids were infected with EV-A71 and Whole Well, Organoid, Cells and Media fractions (Fig. 5a) were collected by differential sedimentation (Methods, Extended Data Fig. 5 and Supplementary Table 4). Samples were subjected repetitive freeze–thaw to release intracellular virus before plaque assay. As expected, inhibition of force sensing by treatment with GsMTx4 did not affect overall viral growth, as evidenced by the Whole Well fraction, although significantly less virus was found in the extruded Cells fraction. The amount of infectious virus within intact organoids was not increased by GsMTx4 treatment, even though the release of infected cells was blocked. Instead, significantly more virus was observed in the Media fraction when extrusion was blocked (Fig. 5i). We surmise that the extrusion of cells from the epithelial layer prevents the outcome that would otherwise occur: the release of cell-free virus.

## Discussion

Infected cells experience a variety of metabolic, oxidative and misfolding stresses that trigger innate cellular responses such as apoptosis, autophagy and the synthesis of inflammatory mediators. Successful viruses inhibit or subvert many of these responses to enhance viral replication. Here we report that EV-A71 also affects mechanosensory signalling pathways. In the polarized cells of colon organoids, EV-A71-infected cells are preferentially extruded into the apical extracellular milieu that corresponds to the colonic lumen. This process could be advantageous to both the host, by eliminating infected cells from the intestinal epithelium, and for the viral population, whose collective extrusion probably facilitates inter-host transmission. In EV-A71-infected mice, shortening of the intestinal villi has been previously observed^37,38^. Villous shortening in other disease contexts results from increased rates of cell loss by extrusion^39^. Therefore, the mechanosensitive expulsion of infected cells from organoids is consistent with, and may explain, these in vivo observations.

In this work, we showed that the extrusion of EV-A71-infected enterocytes from polarized human colonoids is hindered by the small peptide GsMTx4, an inhibitor of mechanosensitive ion channels^33^. These data implicate force sensing as the trigger for extrusion of infected cells. In cultured polarized monolayers and intestinal epithelia, force-sensitive ion channel Piezo1 is known to act as a sensor for cell density homeostasis^29,40^. When epithelia are overcrowded, Piezo1 induces extrusion of live, non-apoptotic cells until homeostatic cell numbers are re-established^29^. Piezo proteins are plasma-membrane-embedded homotrimers, with propeller-like arms and a central Ca^+2^-permeable pore^41^. Upon deformation of the plasma membrane by mechanical force, the arms are thought to reposition, inducing a conformational change in which the pore opens^41^. We postulate that Piezo1 is a likely candidate for the force-driven extrusion of infected cells reported here.

**Extended Data Fig. 6:**
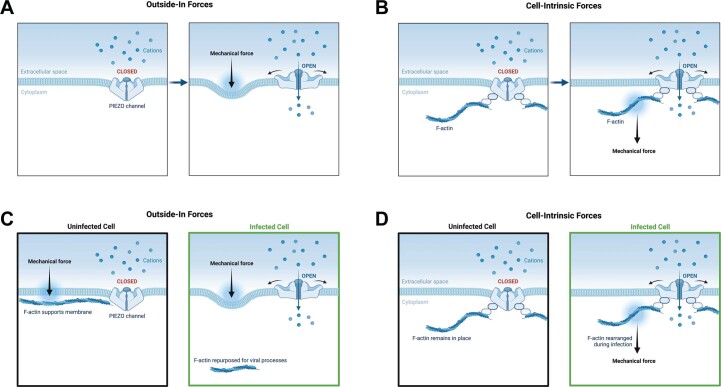
Potential mechanisms of mechanosensitive ion channel activation in infected cells. We provide two hypotheses for potential means of activation of mechanosensitive ion channels in infected cells that may drive infected cell extrusion. Mechanosensitive ion channels such as Piezo1 can be activated by (**a**) external compression forces that induce membrane deformation, as well as (**b**) cell-intrinsic mechanical forces transmitted through tethered cytoskeletal filaments. (**c, d**) We present conceptual models for how viral infection may induce channel activation via both outside-in or cell-intrinsic forces. (**c**) In uninfected cells, cytoskeletal filaments provide structural support beneath the plasma membrane, reducing membrane deformation from cell-extrinsic forces. In infected cells wherein actin is rearranged, this structural support may be missing, resulting in ‘softer’ membranes more easily deformed by external compression forces. (**d**) In the force-through-filament model, cell-intrinsic mechanical forces are transmitted to Piezo channels via tethering to cytoskeletal filaments. In infected cells, cytoskeletal rearrangements may transmit forces directly to tethered Piezo channels, inducing activation. Adapted from ‘PIEZO Channels: How Do They Allow Mechanosensation?’, by BioRender.com (2022). Retrieved from https://app.biorender.com/biorender-templates.

In this study, we observed that the infected cells extruding from human colonoids were rounded and had lost the actin microvilli on their apical surfaces. Contrastingly, infected cells within colonoids appeared similar in size and shape to uninfected neighbours. Enteroviruses are known to cause global re-organization of host cell components including all cytoskeletal elements^42–44^. Indeed, drastic cytoskeletal re-arrangements are major contributors to the oft-used description of the ‘cytopathic effect’ caused by many viruses^45^. Reduction in both membrane tension and cytoskeletal tethering have been implicated in Piezo1 activation^41,46,47^. It is likely that cytoskeletal disruptions induced by viral infection change the biomechanical properties of EV-A71-infected cells, leading to Piezo1 activation and subsequent extrusion (Extended Data Fig. 6).

**Fig. 6:**
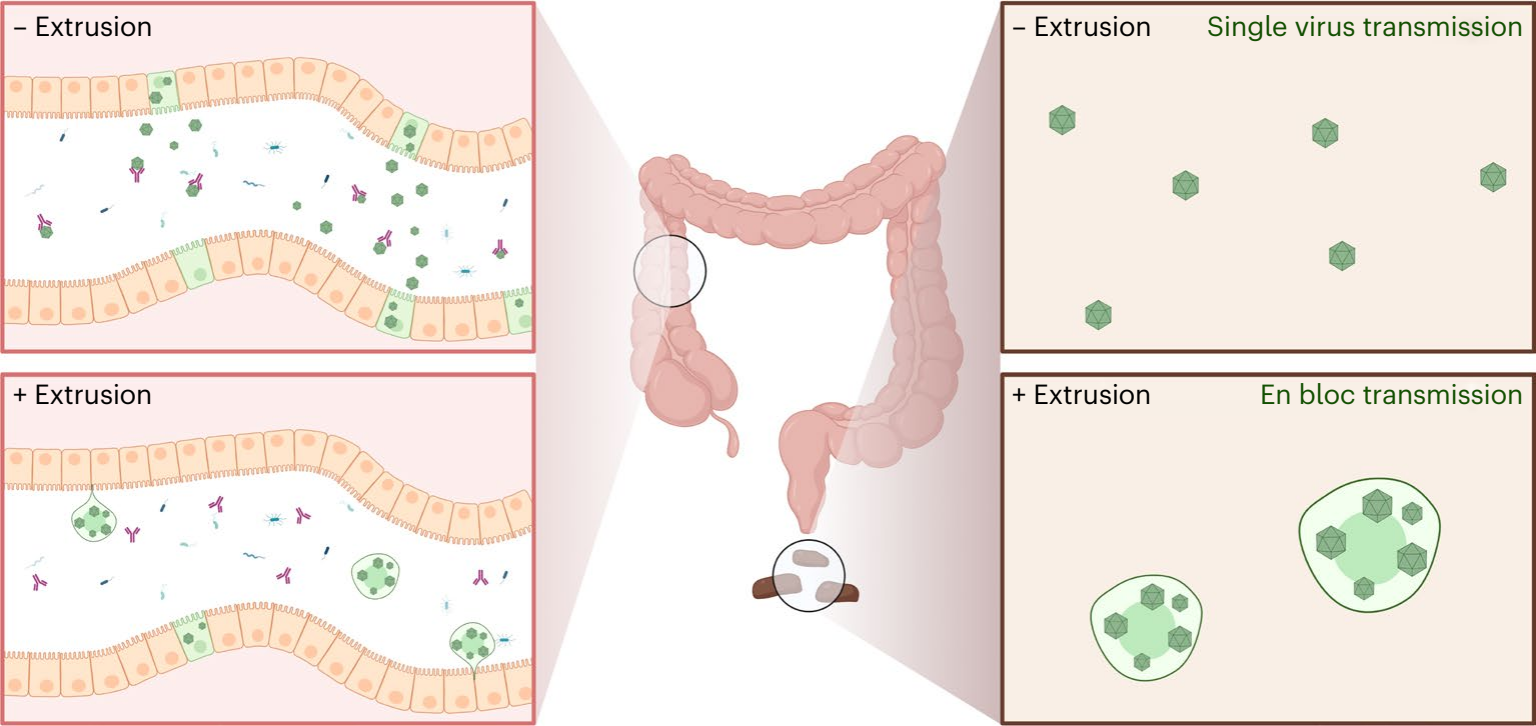
Model for implications of infected cell extrusion on EV-A71 spread. EV-A71-infected cells extruded from the colon into the gastrointestinal lumen may play an integral role in faecal–oral transmission. When cells carrying infectious virus are extruded, progeny virions are expected to transit through the gut and be excreted in stool within live extruded cells. Within cells, virions may be protected from intestinal contents such as mucosal antibodies. Within-cell bundling of virions might additionally facilitate en bloc transmission, allowing for viral genomic diversity to be maintained. The potential for host benefits of infected cell extrusion in vivo such as quicker viral clearance or improved tolerance to infection remain intriguing open questions for further study.

The removal of infected cells by mechanical cell competition might benefit the host by limiting local viral spread in infected tissue. In addition, when extrusion was blocked, an increase in extracellular virus was observed, suggesting that infected cells forced to remain within organoids release virus into the medium through lysis or unconventional secretion. If infected cells are on the verge of lysis, their extrusion could benefit the host by maintaining the epithelial barrier, reducing inflammation, or both (Fig. 6).

Extruded cells could also serve as a means for viral spread from one region of gastrointestinal tissue to a more distal region in the same host, or to another host (Fig. 6). We found that EV-A71-infected cells themselves are infectious to both cell monolayers and previously uninfected organoids. Transiting through the colon within an extruded cell might protect virions from luminal contents such as mucosal antibodies (Fig. 6). Such en bloc viral delivery can have several consequences, due to the transmission of concentrated virus and the maintenance of the complexity of the intracellular viral quasispecies population^5,48–50^.

Our findings add to a growing body of evidence that intracellular pathogens can be removed from epithelial layers through the controlled ejection of infected cells via a variety of mechanisms. Rotavirus, reovirus, respiratory syncytial virus and *Salmonella* all trigger pyroptotic or apoptotic cell death in single infected cells that are subsequently extruded^31,51–54^. In contrast, *Listeria* and measles virus both induce massed shedding, with scores of live infected cells forming large mounds that aggregate atop polarized epithelia^36,55,56^. The unique phenomenon of force-dependent single-cell extrusion of infected cells described here may accompany infection with additional intracellular pathogens that remain to be identified.

In summary, these findings identify the phenomenon of live extrusion of virus-infected cells initiated by mechanosensitive ion-channel activity. Mechanosensitive extrusion may serve a crucial innate immune function by initiating the expulsion of infected cells from epithelial tissue. Furthermore, given that extruded cells can initiate further viral infection, shedding of virus-infected cells may serve as a previously unappreciated means of faecal–oral transmission.

## Methods

### Epithelial organoid cultivation and growth

Organoids were generated following the principles described in ref. ^8^. Gastrointestinal epithelial organoids were generated previously by the lab of Calvin Kuo at Stanford University^13^, through dissection of healthy adult human gastrointestinal tissue biopsies. These were de-identified and obtained by the Stanford Tissue Bank with patient consent and approval by the Stanford University institutional review board. Tissue biopsies were acquired without specifically collecting patient information about age and sex, and no particular targeted or planned enrolment was done (https://www.sciencedirect.com/science/article/pii/S2211124719301457?via%3Dihub).

For maintenance, organoids were seeded within Cultrex Reduced Growth Factor Basement Membrane Matrix, Type II (BME, equivalent to Matrigel) in droplets within a 24-well tissue culture treated plate (40 µl per well). BME was polymerized by incubation for 10 min at 37 °C, then growth medium was overlaid atop BME. Growth medium consists of Advanced Dulbecco’s modified Eagle medium (DMEM)/F12, 1 mM HEPES, 1× Glutamax, 1× B27 (without vitamin A), 1 mM *N*-acetyl-cysteine (for intestinal and colonic samples only), 10 nM gastrin, 50 ng ml^−1^ epidermal growth factor, 10 mM nicotinamide, 500 nM A83-01, 10 µM SB202190, 100 ng ml^−1^ FGF10 (for gastric samples only) and 50% L-WRN-conditioned medium (contains Wnt3a, R-spondin 3 and Noggin). L-WRN-conditioned medium was prepared from L-WRN cells^57^. L-WRN-conditioned medium was aliquoted and frozen at −80 °C for long-term storage with no negative effects on organoid growth observed. Growth medium was replaced every 1–4 days as needed.

To passage, organoids were dissociated to single cells in TrypLE Express for 10–15 min at 37 °C, manually disrupted by pipetting, then trypsin was inactivated with FBS. On ice, cells were filtered through a 70-µm-pore nylon mesh cell strainer to remove large clumps of cells or undissociated organoids. Cells were counted on a Countess II Cell Counter (ThermoFisher) and reseeded in BME at a concentration of 5 × 10^3^ to 1.5 × 10^4^ cells per well. For 2–3 days after initial passage, 10 µM Y27623 and 250 nM CHIR99021 were included in growth medium to prevent detachment-mediated cell death. Organoids were passaged every 4–10 days as needed. All organoids used were tested with Myco-Sniff (MP Biomedicals) to ensure no mycoplasma contamination was present.

### Epithelial organoid differentiation and polarity reversal

After 4–7 days of growth, organoids were removed from Matrigel and induced to revert their polarity in order to expose the apical surface^12^. Organoids were incubated in 5 mM EDTA in phosphate-buffered saline (PBS) at 4 °C for 40 min, washed with DMEM, and resuspended in differentiation medium: Advanced DMEM/F12, 1 mM HEPES, 1× Glutamax, 1× B27, 1 mM *N*-acetyl-cysteine (for intestinal and colonic samples only), 10 nM gastrin, 50 ng ml^−1^ epidermal growth factor, 10 ng ml^−1^ Noggin, 500 nM A83-01, 5 µM γ-Secretase Inhibitor IX (also known as DAPT, colonic samples only), 100 ng ml^−1^ FGF10 (for gastric samples only) and 10 µM Y27623. Organoids in suspension culture were plated in ultralow-attachment plates or flasks (Corning Costar) and incubated at 37 °C for 5 days to complete differentiation and polarity reversal before experimental use.

### RD cells, HeLa cells and virus propagation

RD cells, a gift from the lab of Peter Sarnow, were cultured in DMEM (Hyclone; 4,500 mg l^−1^ glucose, 4 mM l-glutamine and 1 mM sodium pyruvate) supplemented with 10% foetal bovine serum (Omega Life Sciences), 1× non-essential amino acids (MEM NEAA, Gibco) and 1× penicillin–streptomycin (Gibco). RD cells were grown at 37 °C with 5% CO_2_. HeLa cells were cultured in DMEM (Hyclone; 4,500 mg l^−1^ glucose, 4 mM l-glutamine and 1 mM sodium pyruvate) supplemented with 10% foetal bovine serum (Omega Life Sciences) and 1× penicillin–streptomycin (Gibco). Neither RD nor HeLa cells are on the list of known misidentified cell lines maintained by the International Cell Line Authentication Committee (https://iclac.org/databases/cross-contaminations/). Information about authentication of cell lines can be found in Supplementary Information.

EV-A71 strain Taiwan/4643/98 was amplified from an infectious clone produced by the lab of Jen-Ren Wang using RD cells^58^. The full complementary DNA sequence of the virus strain used can be found under GenBank Accession number JN544418. Virus stocks from the second viral passage in RD cells were generated and utilized for infections. Viral titre was determined by plaque assay on RD cells, using a 0.3% (w/v) agarose overlay; plaques were fixed after 3 days with 2% formaldehyde and enumerated by staining with crystal violet. Poliovirus Type 1 Mahoney was amplified from an infectious clone and amplified in HeLa cells. Viral titre was determined by plaque assay on HeLa cells, using a 1.2% (w/v) avicel overlay; plaques were similarly fixed and stained for enumeration after 2 days.

### Viral infections of organoids

**Extended Data Fig. 7:**
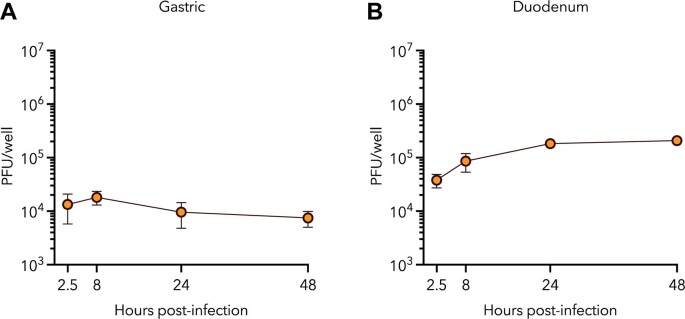
EV71 growth in organoids derived from gastric and duodenal tissue. Apical-out, differentiated epithelial organoids derived from human (**a**) gastric and (**b**) duodenal tissue were infected with Enterovirus A-71. Viral titer was monitored over time by plaque assay. Data from one unique donor and experiment per panel are shown in technical triplicate. Data represented are mean ± SD. Source data

Differentiated, apical-out organoids (5 days post differentiation and polarity reversal) were infected with EV-A71 strain 4643. As RD cells are highly susceptible to EV-A71 infection, multiplicity of infection as defined by RD cells corresponds to a much sparser infection in organoids. Organoids were therefore exposed to a high multiplicity of infection (MOI, 620 PFU per cell) to establish an infection in which five or fewer cells were infected per organoid. Apical-out organoids were prepared from colonic, gastric and duodenal tissues and infected with EV-A71. Between these three tissues, colonoids were the most robustly infected (Fig. 1b and Extended Data Fig. 7).

To separate organoids in suspension from debris before infection, organoids were collected in a 15 ml conical tube and allowed to pellet by gravity on the benchtop (1*g*) for 5–10 min. Supernatant was discarded, and pelleted organoids were washed once in DMEM. Gravity pelleting resulted in very low contamination with single cells (Extended Data Fig. 5 and Supplementary Table 4). An aliquot of this organoid suspension was removed, dissociated with TrypLE Express and counted on Countess II Cell Counter (ThermoFisher) to enumerate cells in organoid suspension for MOI calculations. For experiments in which triplicate infections were performed, organoid suspension was divided into three conical tubes. Organoids were pelleted at 300*g* for 3 min and resuspended in appropriate volume of virus stock (3.7×10^8^ PFU ml^−1^), transferred to an ultralow-attachment plate, and incubated at 37 °C with 5% CO_2_ for 2 h. After incubation, organoids were washed three times in DMEM with centrifugations at 300*g* for 3 min. After the third wash, organoids were resuspended in warm differentiation medium and plated into ultralow-attachment tissue culture plates. Pharmacological treatments were then added if applicable. Infected organoids were incubated at 37 °C with 5% CO_2_ until the experimental endpoint. In experiments during which overall viral titre was quantified, the entire organoid suspension was collected, and the sample was subjected to three repeated freeze–thaw cycles to lyse cells before plaque assay.

For experiments in which differentiated, basolateral-out colonoids were infected with EV-A71, colonoids were grown for 5 days in growth medium, and differentiated for 5 days before infection without removal from BME gel scaffold. On the day of infection, colonoids were removed from the polymerized BME matrix by 40 min incubation in 5 mM EDTA. After washing once with DMEM, colonoids were resuspended in cold differentiation medium containing EV-A71 inoculum (MOI 1,300 PFU per cell), supplemented with 20% (v/v) BME to prevent polarity reversal in suspension culture. Colonoids in the presence of EV-A71 were plated into ultralow-attachment tissue culture plates and incubated for 8 h at 37 °C with 5% CO_2_ before fixation.

For poliovirus infections, differentiated, apical-out ileum and colon organoids were infected using the methodology as described above for EV-A71 infections. Ileum organoids were infected at an MOI of 10 PFU per cell and fixed at 22 hpi. Colon organoids were infected at an MOI of 1 PFU per cell and fixed at 42 hpi.

### Confocal microscopy

Organoids were fixed in 2% paraformaldehyde in 100 mM sodium phosphate buffer (pH 7.4) for at least 30 min and washed with PBS. Organoids were stained by incubating with antibodies and/or stains in blocking/permeabilization buffer (PBS with 3% bovine serum albumin, 1% saponin and 0.02% sodium azide) overnight with gentle agitation. Stained organoids were washed three times in PBS and mounted onto glass slides using Vectashield mounting medium (Vector Laboratories, H-1000), and glass coverslips were affixed using vacuum grease. Organoids were imaged on a LSM 700 confocal microscope (Carl Zeiss) with Zen 2009 software (Carl Zeiss) at 40× or 63× magnification with oil immersion objectives. 3D renderings of organoids were generated using Volocity 3D Image Analysis Software (PerkinElmer version 5.3).

Organoids were stained with 4′,6-diamidino-2-phenylindole dihydrochloride (DAPI; Life Technologies, D1306) and Alexa Fluor 660 phalloidin (Invitrogen, cat. no. A22285) to visualize nuclei and actin. Primary antibody dilutions were performed at the following dilutions: Mouse anti-dsRNA IgG2a Kappa Chain Antibody (J2) SCICONS (acquired by Nordic MUbio) cat. no. 10010200, RRID: AB_2651015 (1:500), Rabbit anti-LIMP2/SCARB2 Recombinant Monoclonal Antibody (22H6L14) ThermoFisher cat. no. 703037; RRID: AB_2734813 (1:100), Rabbit anti-Muc2 Polyclonal Antibody (H-300) SCBT cat. no. sc-15334 RRID: AB_2146667 (1:200) and Rabbit Anti-VIL1 IgG Antibody Sigma Aldrich cat. no. HPA006885; RRID: AB_1080564 (1:100). Secondary antibody dilutions were performed at 1:500 dilution. The following secondary antibodies were used: Goat anti-Mouse IgG (H + L) Cross-Absorbed Secondary Antibody, Alexa Fluor 488 Invitrogen cat. no. A11001; RRID: AB_2534069; Goat anti-Rabbit IgG (H + L) Cross-Adsorbed Secondary Antibody, Alexa Fluor 488 Invitrogen cat. no. A11008; RRID: AB_143165; Goat anti-Mouse IgG (H + L) Cross-Absorbed Secondary Antibody, Alexa Fluor 555 Invitrogen cat. no. A21422; RRID: AB_2535844; Goat anti-Mouse IgG (H + L) Cross-Adsorbed Secondary Antibody, Alexa Fluor 594 Invitrogen cat. no. A11005; RRID: AB_2534073; Goat anti-Rabbit IgG (H + L) Cross-Adsorbed Secondary Antibody, Alexa Fluor 594 Invitrogen cat. no. A11012; RRID: AB_2534079; Rabbit anti-Mouse IgG (H + L) Cross-Adsorbed Secondary Antibody, Alexa Fluor 488 Invitrogen cat. no. A11059; RRID: AB_142495. When multiple secondary antibodies were used in the same organoid staining procedure, secondary antibodies used were raised in the same host species (goat). For visualization of caspase 3/7 activity, CellEvent Caspase 3/7 Green Detection Reagent (Invitrogen, C10723) was added to live organoids at 10 µM after 24 h infection, then organoids were fixed at 48 hpi and stained as described above. Single cells that had been fully extruded from organoids were stained and imaged in the same manner as intact organoids as described above; however, for these experiments a 20× magnification dry objective was used.

### Quantitation of microscopy data

Organoid images were viewed using Volocity 3D Image Analysis Software (PerkinElmer) to gain 3D visualization of each organoid. Cells within organoids that were considered extruding, apoptotic and/or infected were manually counted. An extruding cell was defined by a cell attached to an organoid with a nucleus that has transversed the organoid microvillus brush border. In experiments requiring counting of all cells, the total number of cells in each organoid was enumerated by imaging DAPI at 6 µm *z*-stack intervals to capture the nuclei of each organoid cell in a single *z*-plane. Volocity was used to quantify individual nuclei from these individual *z*-plane images using the 2D Nuclei quantitation feature.

In relevant experiments, quantification of remaining non-extruding or uninfected cells were calculated by subtracting the number of manually counted cells in each group from the total number of cells in each organoid.

In experiments where fully extruded cells were examined, individual cells were identified from 3D renderings of images with *z*-stacks at 1.8 µm intervals. Volocity 3D Image Analysis Software (PerkinElmer) was used to identify individual cells: nuclei were first identified using the Find Objects quantitation feature, object area was increased to surround each nucleus using the Dilate Objects quantitation feature twice iteratively, and signal intensity in each channel was captured using the Measure Objects feature. Objects with a Sum intensity in vRNA channel >2,500 were considered infected.

### Inhibitor treatments

Pharmacological compounds and peptides were tested for their ability to reduce infected cell extrusion. For all experiments, organoids were exposed to compounds after initial viral inoculation and washing steps. Z-VAD-FMK (pan-caspase inhibitor, R&D Systems, 21631) and *para*-nitro-blebbistatin (Myosin II inhibitor, Cayman Chemical, 24171) were solubilized in anhydrous DMSO and added to infected organoids at final concentrations of 100 µM and 50 µM, respectively. GsMTx4 (mechanosensitive ion channel inhibitor, Tocris, 4912/100U) was solubilized in differentiation medium and added to infected organoids at a final concentration of 20 µM. For experiments in which any compounds solubilized in DMSO were included, the final DMSO concentrations in wells were standardized across all conditions at 0.5% (v/v).

### Fractionation of infected organoid cultures

Fractionations of infected organoid suspensions by differential sedimentation in were performed after 8 h of infection. All samples were kept at 4 °C during fractionation. Samples of entire organoid suspension (Whole Well samples) were collected in advance of any sedimentation steps. Next, organoids were pelleted by gravity (1*g*) for 10–15 min. The samples were inspected on a confocal microscope to confirm pelleting of organoids. The pellets containing intact organoids were washed three times in DMEM and finally resuspended in DMEM for future analysis (Organoids samples). Examination of the collected organoids showed limited contamination with single cells (Extended Data Fig. 5 and Supplementary Table 4). The supernatants containing extruded cells in original medium (Cells + Media samples) were further spun at 600*g* for 3 min to generate Cells and Media samples. The cell pellets were similarly washed three times in DMEM and resuspended in DMEM for future analysis (Cells samples); no organoids were observed in these preparations. The supernatants from the 600*g* spin were also collected (Media samples). In experiments with a spike-in of exogenous virus to ensure wash steps were sufficient to remove free virus contamination from cell fractions, 10^6^ PFU of EV-A71 virus stock was added to the Cells + Media samples and wash steps proceeded as described above. All fractions were subjected to three cycles of freezing and thawing to release intracellular virus in cell- or organoid-containing fractions before determination of virus titres by plaque assay.

### Secondary infections of organoids and RD cells with cells extruded from infected organoids

Extruded cells from infected organoids were collected as in fractionation experiments above. Following gravity pelleting to remove organoids, extruded Cells were flowed through a 70 µm nylon mesh cell strainer. In cases where small organoids were observed flowing through 70 µm strainer, these were removed by an additional 10*g* centrifugation for 3 min. During the third (final) wash of extruded cells, cold differentiation medium was used to wash cells. The supernatant of this wash was retained and used as inoculum on parallel infections to monitor virus levels that may be a result of incomplete removal of cell-free virus. The pellet containing extruded cells was resuspended in cold differentiation medium and immediately used as inoculum on new cells or organoids. RD cells secondarily infected were seeded into 24-well plates 1–2 days before infection, washed once with Dulbecco’s phosphate-buffered saline with Ca^+2^ and Mg^+2^ (DPBS++) immediately before infection, inoculated for 1 h at 37 °C with 150 µl wash supernatant or extruded cells, and washed again with DPBS++ before addition of 1 ml per well of RD cell medium. Cell medium was collected and frozen at −20 °C immediately following infection (1 h) or after 16 h infection. Secondary infections of organoids were performed using the methods described above for EV-A71 infections of organoids; however, since the immediate use of extruded cells as inoculum made it impossible to quantify viral titre of inoculums before their use in secondary infections, secondarily infected cells were not counted for determination of MOI before infection.

### Statistics and reproducibility

Statistical data analysis was performed in GraphPad Prism 9. Unless otherwise indicated, all experiments were performed three independent times using multiple distinct organoid donor lines to account for donor-specific differences. In experiments for which data from one representative experiment are shown, experimental findings were reproduced using organoids from an additional donor line. All microscopy images shown are representative of experiments in which a minimum of ten organoids were surveyed with similar results. For quantitative microscopy data in the main text (Figs. 2d,e, 3f and 4f), three independent experiments surveying at least ten organoids each were performed; mean values from independent experiments were used for statistical testing. In microscopy graphs, circles represent measurements of individual organoids (technical replicates) while triangles represent measurements obtained by accumulating data from all organoids from the same experiment (biological replicates). Colour of symbols classify independent experiments. This ‘SuperPlot’ graph format used here has been described in detail by Lord et al.^59^. In all graphs, data are presented as mean ± standard deviation (s.d.). Information about specific statistical tests in each analysis can be found in the figure legends. For Gaussian statistical tests used, data distribution was assumed to be normal but this was not formally tested. Exact *P* values of statistical testing can be found in source data files. No statistical methods were used to pre-determine sample sizes, but our sample sizes are similar to those reported in previous publications^9,10,13^.

Within each independent experiment, samples (organoids) were assigned randomly to experimental groups. To reduce opportunities for operator error, the organization of the presented experimental conditions (for example, plate layout) was not randomized. Collection of viral titre data (counting of plaques) was performed with randomization and with investigator blinding of samples. Due to the time-intensive nature of collection of microscopy data, randomization and blinding were not applied.

### Reporting summary

Further information on research design is available in the Nature Portfolio Reporting Summary linked to this article.

## Supplementary information

Supplementary Information Supplementary Tables 1–4.

Reporting Summary

## Data Availability

No datasets with mandated deposition were generated in this study. All raw graphical data and associated statistical testing have been made available as source data files. Source data are provided with this paper. Other data that support the findings of this study, including raw microscopy image files, are available from the corresponding author upon request.

## Source data

Source Data Fig. 1 Statistical source data.
Source Data Fig. 2 Statistical source data.
Source Data Fig. 3 Statistical source data.
Source Data Fig. 4 Statistical source data.
Source Data Fig. 5 Statistical source data.
Source Data Extended Data Fig./Table 3 Statistical source data.
Source Data Extended Data Fig./Table 4 Statistical source data.
Source Data Extended Data Fig./Table 7 Statistical source data.

## Extended data

are available for this paper at 10.1038/s41564-023-01339-5.

## Code availability

Code is not applicable.
