# Supplementary material for: Mechanosensitive extrusion of Enterovirus A71-infected cells from colonic organoids
Source: Nat Microbiol. 2023 Mar 13;8(4):629–39. doi: 10.1038/s41564-023-01339-5 (PMC10066035; doi:10.1038/s41564-023-01339-5)
Supplement: Supplementary file 1 — Supplementary Tables 1–4. [file 41564_2023_1339_MOESM1_ESM.pdf]

nature microbiology

Article

https://doi.org/10.1038/s41564-023-01339-5

Mechanosensitive extrusion of Enterovirus A71-infected cells from colonic organoids

In the format provided by the authors and unedited

	Infected	Uninfected	% Infected
Extruding	27	79	25.5%
Nonextruding	41	10,979	0.37%
% Extruding	39.7%	0.71%	

Fisher's exact test, ****P < 0.0001

Supplementary Table 1: Related to Figure 2D-E. 2x2 contingency table displays the number of cells in groups represented in Fig. 2D-E. Cell numbers are summed from three experiments. Two-sided Fisher's exact test, $P < 0.0001$.

	Infected	Uninfected	% Infected
5h	2	8150	0.025%
7h	11	7385	0.149%
9h	3	6439	0.047%

Chi squared test, **P* = 0.0106

Supplementary Table 2: Related to Extended Data Figure 3. Infected cells accumulate in organoids after 7 h infection and diminish after 9 h. A peak in the proportion of infected cells within organoids emerged at 7 hpi and was reduced after 9 hpi. Total numbers of infected and uninfected cells represented in Extended Data Fig. 3A; **P* = 0.0106, Chi squared test, two-sided.

Extruding cells

	Apoptotic	Non-apoptotic	% Apoptotic
Infected	8	41	16.3%
Uninfected	54	58	48.2%
% Infected	12.9%	41.4%	

Fisher's exact test, ****P* = 0.0001

Supplementary Table 3: Related to Figure 3. EV-A71-infected cell extrusion is not driven by apoptosis. 2x2 contingency table displays the number of cells in each group depicted in Fig. 2F. Cell numbers are summed from all three independent experiments. Fisher's exact test, two-sided; *P* = 0.0001.

Fields of View	Organoids Counted	Estimated cells in organoids	Single Cells Counted	Organoid cells per Single Cell
1	224	105,151	60	1,753
2	58	27,227	32	851
3	88	41,309	30	1,377
4	110	51,637	24	2,152
5	62	29,104	23	1,265
Average	108	50,886	34	1,479

Supplementary Table 4: Related to Extended Data Figure 5. Quantification of the *Organoid* fraction of suspension organoid cultures. Intact organoids and single cells were counted from images of five fields of view as shown in Extended Data Figure 5. In three separate experiments (shown in Fig 2D-F), the number of cells per organoid was previously enumerated using fluorescence microscopy, with an average ± SD of 469 ± 102 cells per organoid. Using this value, we estimated the number of cells present within intact organoids in each field of view to be approximately 50,000 while the average field of view contained 34 individual single cells. Therefore, in the *Organoid* fraction, cells within organoids outnumber single cells at a ratio of approximately 1,500 to 1.
