# Supplementary material for: Mechanosensitive extrusion of Enterovirus A71-infected cells from colonic organoids
Source: Nat Microbiol. 2023 Mar 13;8(4):629–39. doi: 10.1038/s41564-023-01339-5 (PMC10066035; doi:10.1038/s41564-023-01339-5)
Supplement: Supplementary file 2 — Reporting Summary [file 41564_2023_1339_MOESM2_ESM.pdf]

nature portfolio

Corresponding author(s): Kirkegaard, Karla

Last updated by author(s): Jan 14, 2023

Reporting Summary

Nature Portfolio wishes to improve the reproducibility of the work that we publish. This form provides structure for consistency and transparency in reporting. For further information on Nature Portfolio policies, see our Editorial Policies and the Editorial Policy Checklist.

Statistics

For all statistical analyses, confirm that the following items are present in the figure legend, table legend, main text, or Methods section.

n/a	Confirmed	
☐	☒	The exact sample size (*n*) for each experimental group/condition, given as a discrete number and unit of measurement
☐	☒	A statement on whether measurements were taken from distinct samples or whether the same sample was measured repeatedly
☐	☒	The statistical test(s) used AND whether they are one- or two-sided<br>*Only common tests should be described solely by name; describe more complex techniques in the Methods section.*
☐	☒	A description of all covariates tested
☐	☒	A description of any assumptions or corrections, such as tests of normality and adjustment for multiple comparisons
☐	☒	A full description of the statistical parameters including central tendency (e.g. means) or other basic estimates (e.g. regression coefficient) AND variation (e.g. standard deviation) or associated estimates of uncertainty (e.g. confidence intervals)
☐	☒	For null hypothesis testing, the test statistic (e.g. *F*, *t*, *r*) with confidence intervals, effect sizes, degrees of freedom and *P* value noted<br>*Give P values as exact values whenever suitable.*
☒	☐	For Bayesian analysis, information on the choice of priors and Markov chain Monte Carlo settings
☒	☐	For hierarchical and complex designs, identification of the appropriate level for tests and full reporting of outcomes
☒	☐	Estimates of effect sizes (e.g. Cohen's *d*, Pearson's *r*), indicating how they were calculated

Our web collection on statistics for biologists contains articles on many of the points above.

Software and code

Policy information about availability of computer code

Data collection	Collection of microscopy data utilized the Zen 2009 software (Carl Zeiss).
Data analysis	Quantification of caspase activation in fully extruded cells, as well as enumeration of all cells within organoids was achieved using Volocity 3D Image Analysis Software (Perkin-Elmer version 5.3). All statistical testing was performed using GraphPad Prism 9.

For manuscripts utilizing custom algorithms or software that are central to the research but not yet described in published literature, software must be made available to editors and reviewers. We strongly encourage code deposition in a community repository (e.g. GitHub). See the Nature Portfolio guidelines for submitting code & software for further information.

Data

Policy information about availability of data

All manuscripts must include a data availability statement. This statement should provide the following information, where applicable:

- Accession codes, unique identifiers, or web links for publicly available datasets
- A description of any restrictions on data availability
- For clinical datasets or third party data, please ensure that the statement adheres to our policy

No datasets with mandated deposition were generated in this study. All raw graphical data and associated statistical testing have been made available as Source Data files. Other data that support the findings of this study, including raw microscopy image files, are available from the corresponding author upon request, due

to the large storage size of such files. This study used or referenced the following publicly available dataset: the genomic sequence of the EV-A71 Taiwan/4643/98 strain used in this study can be found under GenBank Accession number JN544418.

Human research participants

Policy information about studies involving human research participants and Sex and Gender in Research.

Reporting on sex and gender	*Use the terms sex (biological attribute) and gender (shaped by social and cultural circumstances) carefully in order to avoid confusing both terms. Indicate if findings apply to only one sex or gender; describe whether sex and gender were considered in study design whether sex and/or gender was determined based on self-reporting or assigned and methods used. Provide in the source data disaggregated sex and gender data where this information has been collected, and consent has been obtained for sharing of individual-level data; provide overall numbers in this Reporting Summary. Please state if this information has not been collected. Report sex- and gender-based analyses where performed, justify reasons for lack of sex- and gender-based analysis.*
Population characteristics	*Describe the covariate-relevant population characteristics of the human research participants (e.g. age, genotypic information, past and current diagnosis and treatment categories). If you filled out the behavioural & social sciences study design questions and have nothing to add here, write "See above."*
Recruitment	*Describe how participants were recruited. Outline any potential self-selection bias or other biases that may be present and how these are likely to impact results.*
Ethics oversight	*Identify the organization(s) that approved the study protocol.*

Note that full information on the approval of the study protocol must also be provided in the manuscript.

Field-specific reporting

Please select the one below that is the best fit for your research. If you are not sure, read the appropriate sections before making your selection.

☒ Life sciences ☐ Behavioural & social sciences ☐ Ecological, evolutionary & environmental sciences

For a reference copy of the document with all sections, see nature.com/documents/nr-reporting-summary-flat.pdf

Life sciences study design

All studies must disclose on these points even when the disclosure is negative.

Sample size	For each experiment type, an appropriate sample size and number of data points were collected. For example, in microscopy experiments N = 3 separate experiments are included, with a minimum of 10 organoids included in each measurement. In viral titer experiments, N = 3 independent infections were performed in triplicate. No statistical methods were used to pre-determine sample sizes but our sample sizes are similar to those reported in previous publications, as cited in the text.
Data exclusions	No relevant data were excluded
Replication	All experiments were replicated to ensure reproducibility. To address differences arising from organoid donor lines, multiple donor lines were used to repeat experiments. As such, in experiments where data is shown for N = 3 independent experiments, two experiments were performed using organoids from the same donor line and a third experiment used organoids from an additional donor line. In viral titer experiments in which independent infections were performed in triplicate, all findings were successfully reproduced in another experiment using organoids from a second unique donor line.
Randomization	Within each independent experiment, samples (organoids) were assigned randomly to experimental groups. To reduce opportunities for operator error, the organization of the presented experimental conditions (e.g. plate layout) was not randomized.
Blinding	Investigators were blinded to experimental sample during plaque enumeration for quantification of viral titer. Due to the time-intensive nature of collection of microscopy data, randomization and blinding were not applied.

Reporting for specific materials, systems and methods

We require information from authors about some types of materials, experimental systems and methods used in many studies. Here, indicate whether each material, system or method listed is relevant to your study. If you are not sure if a list item applies to your research, read the appropriate section before selecting a response.

Materials & experimental systems

n/a	Involved in the study	
☐	☒	Antibodies
☐	☒	Eukaryotic cell lines
☒	☐	Palaeontology and archaeology
☒	☐	Animals and other organisms
☒	☐	Clinical data
☒	☐	Dual use research of concern

Methods

n/a	Involved in the study	
☒	☐	ChIP-seq
☒	☐	Flow cytometry
☒	☐	MRI-based neuroimaging

Antibodies

Antibodies used	Rabbit anti-Muc2 Polyclonal Antibody (H-300) SCBT Cat# sc-15334; RRID: AB_2146667<br>Rabbit anti-LIMP2/SCARB2 Recombinant Monoclonal Antibody (22H6L14) ThermoFisher Cat# 703037; RRID: AB_2734813<br>Mouse anti-dsRNA IgG2a Kappa Chain Antibody (J2) SCICONS (Acquired by Nordic MUbio) Cat# 10010200, RRID: AB_2651015<br>Rabbit Anti-VIL1 IgG Antibody Sigma Aldrich Cat# HPA006885; RRID: AB_1080564<br>Goat anti-Mouse IgG (H+L) Cross-Absorbed Secondary Antibody, Alexa Fluor 488 Invitrogen Cat# A11001; RRID: AB_2534069<br>Goat anti-Rabbit IgG (H+L) Cross-Adsorbed Secondary Antibody, Alexa Fluor 488 Invitrogen Cat# A11008; RRID: AB_143165<br>Goat anti-Mouse IgG (H+L) Cross-Absorbed Secondary Antibody, Alexa Fluor 555 Invitrogen Cat# A21422; RRID: AB_2535844<br>Goat anti-Mouse IgG (H+L) Cross-Adsorbed Secondary Antibody, Alexa Fluor 594 Invitrogen Cat# A11005; RRID: AB_2534073<br>Goat anti-Rabbit IgG (H+L) Cross-Adsorbed Secondary Antibody, Alexa Fluor 594 Invitrogen Cat# A11012; RRID: AB_2534079<br>Rabbit anti-Mouse IgG (H+L) Cross-Adsorbed Secondary Antibody, Alexa Fluor 488 Invitrogen Cat# A11059; RRID: AB_142495
Validation	Primary antibodies validated by provider:<br>Rabbit anti-Muc2: Validated by IF subcellular localization.<br>Rabbit anti-LIMP2/SCARB2: Validated by co-localization with lysosomes.<br>Mouse anti-dsRNA: Validated by IF in presence and absence of viral infections.<br>Rabbit Anti-VIL1: Validated by IF subcellular co-localization with plasma membrane and IHC with tissue colocalization with a separate anti-VIL1 Ab<br><br>Commercial Invitrogen IgG (H+L) Cross-Absorbed Secondary Antibodies have been validated extensively by the provider and in the literature by Flow, WB, IF, and IHC, and were also validated by our research group using experimental no-primary controls.

Eukaryotic cell lines

Policy information about cell lines and Sex and Gender in Research

Cell line source(s)	L-WRN cells ATCC Cat# CRL-3276 were provided by the lab of Calvin Kuo.<br>Organoid lines were previously generated and provided by the lab of Calvin Kuo.<br>RD cells ATCC Cat# CCL-136 were provided by the lab of Peter Sarnow.<br>HeLa cells ATCC Cat# CCL-2 were provided by Emma Abernathy.
Authentication	RD and HeLa cells: cellular morphology, plaque morphology, and viral permissiveness was consistent with literature observations.<br>Organoid lines: evaluated by qPCR and IF for expression of colonic and ileal tissue-specific genes by Julia Co, et al. 2019. Cell Reports.
Mycoplasma contamination	All cell lines tested negative for mycoplasma contamination.
Commonly misidentified lines<br>(See ICLAC register)	No commonly misidentified cell lines were used in this study.
